# A Revised Version of Diabetes Quality of Life Instrument Maintaining Domains for Satisfaction, Impact, and Worry

**DOI:** 10.1155/2018/5804687

**Published:** 2018-07-22

**Authors:** Mohamad Adam Bujang, Tassha Hilda Adnan, Nur Khairul Bariyyah Mohd Hatta, Mastura Ismail, Chien Joo Lim

**Affiliations:** ^1^Clinical Research Centre, Sarawak General Hospital, Ministry of Health Malaysia, Kuching, Malaysia; ^2^National Clinical Research Centre, Ministry of Health Malaysia, Kuala Lumpur, Malaysia; ^3^Health Clinic Seremban 2, Ministry of Health Malaysia, Seremban Negeri Sembilan, Malaysia

## Abstract

**Background:**

Diabetes quality of life (DQoL) instrument has been widely used to measure quality of life among diabetes patients. This study aimed to develop a revised version of DQoL instrument that incorporated issues of redundancies in the items and strengthen the basis of validity of the instrument.

**Methods:**

This was a cross-sectional study where diabetes patients were recruited from December 1, 2014, until end of March 2015 at a public health clinic in Peninsular Malaysia. A questionnaire that included patients' information and DQoL instrument was distributed to patients. Item selection of DQoL instrument was conducted to screen and finalize the items based on issues of missing values and redundancy. Validity testing was conducted for the revised DQoL instrument based on exploratory factor analysis, confirmatory factor analysis, and Rasch analysis.

**Results:**

The pattern structure matrix yielded three domains similar to the original version with 18 items. The minimum factor loading from the structure matrix was 0.358. The item's and person's reliability was excellent with 0.92 and 0.84 for “satisfaction” domain, 0.98 and 0.60 for “impact” domain, and 0.99 and 0.57 for “worry” domain, respectively. Confirmatory factor analysis has dropped 5 items and the revised version of DQoL contained 13 items. Composite reliability of the revised version was computed for “satisfaction” domain (0.922; 95% CI: 0.909–0.936), “impact” domain (0.781; 95% CI: 0.745–0.818), and “worry” domain (0.794; 95% CI: 0.755–0.832).

**Conclusion:**

A revised version of DQoL that maintains the conceptualization of “satisfaction,” “impact,” and “worry” with 13 items was successfully developed.

## 1. Introduction

Diabetes quality of life (DQoL) instrument was published in 1988 by the Diabetes Control and Complications Trial (DCCT) Research Group [1]. It was initially developed for a multicenter controlled trial to investigate the effect of two different diabetes treatment interventions on the incidence and progression of early vascular complications. The DQoL instrument which contained 46 items was used to measure health-related quality of life among diabetes patients based on three main domains, namely, “satisfaction,” “impact,” and “worry.” This instrument has been widely used in diabetes research for decades.

As a questionnaire to measure quality of life for patients with diabetes mellitus, DQoL was reported to have very strong reliability and proven to be valid [1, 2]. Its reliability measures were evaluated based on test-retest reliability and internal consistency. Meanwhile, the basis of its validity was supported based on content and concurrent validity. Content validity was conducted among a group of experts while the concurrent validity was determined by the support of other questionnaires such as Symptom Checklist-90-R (SCL), the Bradburn Affect Balance Scale (ABS), and the Psychosocial Adjustment to Illness Scale (PAIS). However, the development of DQoL instrument was not supported by construct validity, although statistical technique such as exploratory factor analysis is a common type of analysis which can be applied in questionnaire development to construct domains for a latent variable [1].

The instrument has been translated and validated into various languages such as Spanish, Turkish, Japanese, Chinese, Taiwanese, Iranian, and Malaysian [3–9]. Some studies showed improved evidence on the validity of DQoL instrument by testing the association of DQoL instrument and diabetes complications [7]. Proven its stability and validity, DQoL instrument is widely used for diabetes research despite its limitations and is still relevant until now.

Initially, the foundation of DQoL instrument is supported by theory of the conceptual scheme of domains and variables in a quality of life assessment [10]. Perhaps one of the limitations for DQoL instrument is that many items are required to represent the three main domains; nonetheless, it is necessary to ensure that various perspectives of quality of life among diabetes patients are well covered. Other well-known quality of life instruments also have a relatively large number of items with at least 30 items [11–13].

An important consideration among diabetes patients is that there are substantial number of elderly patients with varying severity of diabetes-related complications. A questionnaire with substantial items will require more time to be completed. Invalid responses may occur due to quick responses without proper thinking and evaluation on every item by the respondents. A high number of missing values are likely to happen, consequently leading to frustration to the researcher as items were not responded properly. Hence, developing a shorter version of DQoL instrument is crucial.

Some efforts have been made to develop a shorter version of DQoL instrument, and it was considered as a brief version of DQoL instrument. However, the function of the three main domains was not covered [14]. Therefore, the intention of our study is to develop a newly revised instrument for DQoL while maintaining the assessment of the three main domains, namely, “satisfaction,” “impact,” and “worry.” The initial concept of the domains is in line with the requirement of a quality of life measure [10]. Besides the content, the three main domains need to be validated statistically. Therefore, the aims of this study were to introduce the revised version of DQoL instrument and to support the validity with quantitative measures using exploratory factor analysis, confirmatory factor analysis, and Rasch analysis.

## 2. Methods

The methods were divided into three sections, namely, study design, item selection, and proof of the validity basis based on statistical measures.

### 2.1. Study Design

This was a cross-sectional study where type 2 diabetes patients were recruited from December 1, 2014, until end of March 2015 at a government health clinic (Health Clinic Seremban 2) in Malaysia. The validated revised DQoL instruments [9] were distributed to diabetes patients attending their follow-up visit. Besides DQoL instrument, other information such as demographic profiles and clinical variables were also collected and analyzed. Subjects' clinical information were obtained from their health record. Written consent was obtained from all patients before their participation in the study. Ethical approval was received on July 18, 2014, from the Medical Research and Ethics Committee (MREC) of Ministry of Health Malaysia (NMRR ID: NMRR-14-522-19377).

### 2.2. Item Selection

The original DQoL instrument has 46 items with 15, 20, and 11 items for each domain of “satisfaction,” “impact,” and “worry.” In order to confirm the construct of the three domains, the revised version of DQoL instrument needs to be tested with construct validity such as exploratory factor analysis and confirmatory factor analysis. This was to confirm the ability of the revised version of DQoL instrument in measuring the construct intended as well as the content validity. Besides that, the construct validity also aimed to prove that the shorter version has an appropriate set of relevant items reflecting the full content of the domains being measured.

The development of the revised version started with selection of items. Items were assessed based on two criteria: missing value of the response less than 10.0% and no duplication in terms of the content. Evaluation of missing values is important to identify and omit irrelevant items. Issue of duplication should also be taken into consideration as duplicate items may affect the construct of the domains, and cross-loading between items may occur. Only one item was retained once duplication was identified, and priority was given based on the suitability of the item reflecting the definition of the domain.

### 2.3. The Proof of Validity Basis for a Newly Modified Version of DQoL Instrument

Exploratory factor analysis was performed to determine the underlying relationship between the constructs of DQoL domains. The scale of reverse scoring items (I8 and I16) was transformed before analysis was conducted [15]. Moderate correlation was expected between the domains, and hence, extraction method “principle axis factors” and rotation method “promax” were chosen. The approach of analysis was as the following:

1. Exploratory factor analysis was first conducted on individual domain to make sure all the items were stable in their respective domain. If the number of domain produced was more than one, only the domain with largest number of items would be chosen.
2. Subsequently, exploratory factor analysis was tested for all the items (no further process took place because the construct of the domains was successful. All the items fell under their respective domains).

Person's and item's reliability was analyzed using Rasch analysis while confirmatory factor analysis was conducted to examine how well the measured variables represent the number of constructs. Rasch analysis, introduced by George Rasch (1960), is a powerful tool to develop and validate an instrument [16]. Classically, internal consistency index like Cronbach's alpha can be used to assess the performance of a scale. However, the measurement can be misleading, as high correlation among a subset of items may be due to the similarity of wordings rather than the relationship of items with the construct in question [17]. Hence, high alpha values might therefore be indicative of an inferior rather than superior quality of the scale [18]. The Rasch analysis has been used to construct new questionnaires and was extensively used to review and improve existing questionnaires that were constructed using Likert scales. Hence, this study assessed and simplified the DQoL questionnaire using Rasch analysis to optimize item that fits to the construct, and the psychometric properties were measured with fit statistics and separation indices. Reliability and empirical validity of the simplified/revised version were analyzed further and compared with those of the full/original version.

Another fundamental element of measurement: unidimensionality was used to examine whether the items formed one common underlying latent variable. Unidimensionality was assessed using the fit statistics and principal component analysis (PCA) of the residuals. Both infit and outfit statistics, measured as mean square standardized residuals (MNSQ), indicated how well items fit into the underlying construct. The cut-off range for MNSQ from 0.5 to 1.5 could be considered productive for measurement [19]. Unidimensionality was further assessed by principal component analysis (PCA) of the residuals, using two criteria. Firstly, the variance explained by the measurement dimension is large, at least 40% [20], and the empirical calculation should be similar to the model [21]. Secondly, the first contrasts in the residuals reporting the unexplained variance by the principal component should be small, that is, not more than 15% [22]. Therefore, to strengthen the evidence of the construct, Rasch analysis was applied to evaluate the new construct and compare it with the original construct.

The model was further tested using confirmatory factor analysis and model fit depending on absolute fit using standardized root mean square residual (SRMR), parsimony correction fit index using root mean square of approximation (RMSEA), comparative fit indices using comparative fit index (CFI), and Tucker-Lewis index (TLI). If the model did not fit the data, necessary modifications to improve model fit were done by removing items with low factor loading, high standardized residuals, and high modification index. Modifications were done until the model was reasonably fit as well as theoretically sound. Convergent validity (reliability) of the measurement model was checked using composite reliability by Raykov's procedure. This method would yield more accurate reliability estimation where correlation term on error was added.

All analyses were conducted using SPSS (IBM Corp. Released 2012. IBM SPSS Statistics for Windows, Version 21.0. Armonk, NY: IBM Corp.), Mplus (Muthén, L. K., & Muthén, B. O., 1998–2011), and Winstep (Linacre, J. M. (2016). Winsteps® Rasch measurement computer program. Beaverton, Oregon: Winsteps.com).

## 3. Results

### 3.1. Demographic and Clinical Profile of Patients

A total of 536 patients have participated in the study. Majority were females (53.7%), of Malay race (55.8%) and married (84.3%). The mean (SD) age was 56.7 (11.2) years. About 68.1% of the participants have hypertension and 45.7% of them have dyslipidemia. Some of the participants have experienced diabetes complications such as retinopathy (5.2%), nephropathy (3.4%), neuropathy (1.1%), and cardiovascular (4.7%).

### 3.2. Item Selection

For “satisfaction” domain, eight items were dropped out of 15 items. Two items were dropped due to missing values more than 10.0%. For example, satisfaction item 10 (S10), concerning satisfaction with sexual life, it was removed as sexual issues is still considered sensitive in Malaysia and many people are uncomfortable to answer such question thus could lead to missing responses. Six items were dropped due to issue of redundancy with items under “impact” domain such as items concerning food intake (S5 and I9) and burden to family members (S6 and I5). Hence, the items retained under “satisfaction” domain were S1, S2, S3, S4, S7, S12, and S15 (Table 1).

For “impact” domain, seven items were dropped out of 20 items. Items I10, I11, and I20 were dropped due to high percentage of missing values (>10.0%). Item I3 (has low blood sugar) was omitted. Item I1 was suitable to represent item I3 if the patients felt sick or had pain due to diabetes treatment. I14 was similar to I16 and both were dropped as they had less impact in terms of physical and psychological. Items I9 and I19 were comparable, and thus, I9 was chosen since it was simpler and more straightforward. Finally, items I1, I2, I4, I5, I6, I7, I8, I9, I12, I13, I15, I17, and I18 were selected for further evaluation (Table 1).

For “worry” domain, eight items were dropped out of 11 items. All the eight items were dropped due to missing values more than 10.0%. Hence, only W8, W9, and W10 were remained for further evaluation (Table 1).

### 3.3. Validity of a Newly Revised Version of DQoL Instrument

Results of exploratory factor analysis by each domain showed that only “impact” domain yielded two factors, whereas the rest only yielded one factor solution. Item 8 in “impact” domain was dropped since its communality value was less than 0.2 and produced factor loading with a negative value. Exploratory analysis was conducted again for 12 items (I1, I2, I4, I5, I6, I7, I9, I12, I13, I15, I17, and I18). Two factors were constructed where factor one had eight items and factor two had four items. Thus, the factor with more items (I1, I2, I4, I5, I6, I7, I9, and I18) was selected for further evaluation.

Exploratory factor analysis was further conducted for the 18 items where seven items were under “satisfaction” domain, eight items under “impact” domain, and three items under “worry” domain. Results showed that the construct produced three domains, and all the items fell under the respective domains. The minimum coefficient of factor loading from the structure matrix was 0.358 (Table 2). The item's and person's reliability was excellent with 0.92 and 0.84, respectively, for “satisfaction” domain, 0.98 and 0.60, respectively, for “impact” domain, and 0.99 and 0.57, respectively, for “worry” domain (Table 3).

### 3.4. Evaluation Based on Rasch Analysis

We subjected the domains and five-point Likert scale to Rasch analysis in a similar manner to that of the full version of the DQoL instrument. The overall performance of both versions for “satisfaction” domain was acceptable with satisfactory fit of high internal consistency to the Rasch model (full version: item separation = 6.01 and reliability = 0.97 and person separation = 2.91 and reliability = 0.89, Cronbach's *α* = 0.95; simplified version: item separation = 3.44 and reliability = 0.92 and person separation = 2.27 and reliability 0.84, Cronbach's *α* = 0.93) (Table 3). Similar to the full version, the person separation and reliabilities were poor for both the domains: “impact” and “worry.” The person separation and reliability were low (<2.0 and <0.8, resp.), with a relevant person sample, indicating the poor discriminatory ability of the simplified/revised DQoL instrument. This implies that the domains may be less sensitive in distinguishing between those perceiving high and low degree of frequency, and that more items may be needed, although a longer questionnaire would be less desirable.

Table 4 shows that all items fit a single overall construct for the simplified/revised version (infit range 0.78–1.27; outfit range 0.70–1.25). In addition to item fit statistics, unidimensionality was assessed further using principal component analysis (PCA) of the residuals. In the simplified/revised DQoL instrument, the variance explained by the measures for the empirical calculation was almost identical to the model (“satisfaction”: 63.3% and 63.5%, respectively; “impact”: 51.9% and 52.4%; “worry”: 62.6% and 62.4%). The first residual factor in the “satisfaction” domain had an eigenvalue of 1.6, representing 9.8% of the unexplained variance, “impact” domain with 1.6 eigenvalue units (19.0%), and “worry” domain with 1.7 eigenvalue units (20.6%). These results suggested unidimensionality of the scale (Table 3).

### 3.5. Evaluation Based on Confirmatory Factor Analysis

Confirmatory factor analysis was performed, and only items with factor loading 0.6 or higher were recruited to ensure all the items recruited explained the domains sufficiently. Item S12 (0.523), I2 (0.526), I6 (0.584), I9 (0.561), and I18 (0.466) were removed. Composite reliability for each domain was computed as well; “satisfaction” domain showed highest composite reliability of 0.922, followed by “worry” domain (0.794) and “impact” domain (0.781). The measurement model for DQoL questionnaire was finalized with 13 items, with six items for “satisfaction” domain, four items for “impact” domain, and three items for “worry” domain, respectively (Table 5).

**Figure 1:**
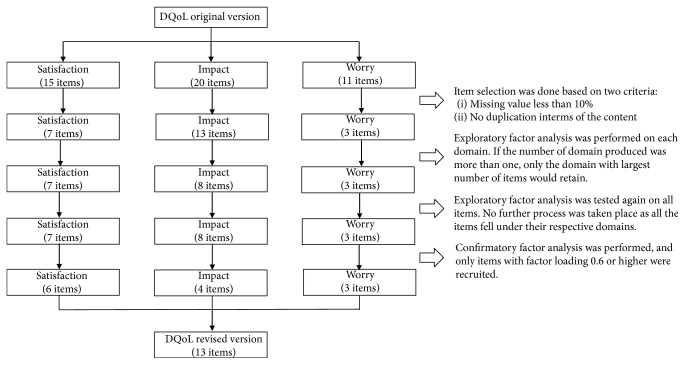
Flow of data analysis.

Analysis was further conducted to examine model fit for both original (46 items) and the revised version (13 items). Both measurement models were compared based on the indices. The original version with 46 items did not fit the cut-off value for all the indices while the revised version with 13 items showed good fit for the measurement model. The *p* value CF fit for the revised version with 13 items was 0.528, indicating no difference between observed data and the specified model (Table 6). The flow of data analysis is summarized in Figure 1.

## 4. Discussion

The revision of the original version of DQoL instrument is crucial especially when there are claims that the instrument is less sensitive to measure quality of life among diabetes patients [14, 23]. The claim is due to the findings from the DCCT group when they first used the instrument and concluded that the DQoL instrument failed to detect any significant differences regarding the association of quality of life towards various clinical outcomes such as status of glycemic control, status of severe hypoglycemia, and status of progression of late complications [2]. This might be due to the fact that quality of life is also influenced by various factors such as marital status, social relationships, the existence of other health problems, patient knowledge, treatment satisfaction, and perceived ability to control one's disease [24–27].

Apart from the external factors mentioned earlier, the original DQoL instrument has a major limitation where the construct of this instrument was not supported by factor analysis. There was no study that validated DQoL successfully using exploratory factor analysis including our data so far. This could be due to some of the items that are not be suitable for majority of the diabetic patients. For instance, a question with regard to sexual life is quite sensitive for some countries [28] and thus leads to a more protective response towards the question. In addition, this item may be irrelevant for patients who are not sexually active. Apart from that, there were also redundancies among the items especially between “satisfaction” and “impact” domains. Therefore, the idea to come out with the revised version was to overcome these issues.

Ideally, all the items should be relevant as a proxy to measure quality of life. However, a well-constructed instrument should be supported by the statistical evidence besides the content alone. Some analyses of validation had been conducted for DQoL instrument [1–9], but it was insufficient since all the effort to prove the validity of current DQoL instrument based on exploratory factor analysis had failed. As exploratory factor analysis is the most common method to validate a quality of life instrument [11–13], it is necessary to reevaluate the items and retest the validity of the revised version of DQoL instrument. It is also crucial to strengthen and improve the stability of the DQoL questionnaire to be used for diabetes research.

The process to validate the current DQoL instrument was successful after few items were omitted. Justifications to omit the items were based on the percentage of missing values and issue of duplication. The items with high percentage of missing values might indicate that the items were irrelevant for most of the diabetic patients. Majority of the items with high missing values were from “worry” domain. For example, items such as whether you will marry or get children are irrelevant for patients who are already married and have children. These items will lead to missing values if they are retained, and subsequently, the total score by domain will be difficult to compute.

Some items were found to have similar meaning especially from domains of “satisfaction” and “impact.” For example, items S6 and I5 were redundant in terms of meaning since both represent the extent of burden of diabetes which contributes to the family members. Individual item should only represent a single construct [29]. Hence, priority was given to impact domain when redundancy was detected between these two domains. The decision was done with consideration that the items reflect more in terms of the impact from diabetes.

For the revised version of DQoL instrument, we proposed the score for each domain and the total score to be converted to percentage as presented in Table 7. Any missing value, although it is unlikely to happen, can be substituted with the median score based on the response from the respective domain if the missing value is not more than one. If missing value is more than one in each domain, the domain score and the total score cannot be calculated based on the proposed rule. The original DQoL instrument allows more than one missing value in calculating a score of the domain [15]. However, in order to maintain the validity of the interpretation especially when the number of items in each domain had been reduced more than 50.0%, only one missing value is allowed for each domain.

Result from exploratory factor analysis showed that the revised version of DQoL instrument has stable and strong construct. The validation was conducted among more than 500 patients in which the sample size was more than enough to conduct an exploratory factor analysis for 18 or even 46 items [30]. In other words, besides content validity, the conceptualization of the three domains was successfully validated. A sufficient sample size in addition to the fact that sufficient sample is needed to conduct factor analysis and to capture an accurate pattern of the responses from the respondents in the intended population is presented [31, 32]. The findings were also supported by Rasch analysis. The item's and person's reliability has improved compared with the original version. In addition, the infit and outfit of each and every item were also within the acceptable range.

The final model for confirmatory factor analysis consisted of 13 items with three factors fit well after respecification. The present study confirmed the validity for the three factors' structure measurement model based on CF fit, RMSEA, SRMR, and TLI, providing a foundation for future study to be conducted to measure quality of life among diabetes patients in Malaysia. The composite reliability estimates proved the good construct validity of present study. Therefore, the evidence from the confirmatory factor analysis and Rasch analysis helped to strengthen the validity of the revised version of DQoL instrument.

Besides an excellent construct, the major strength with the revised version of DQoL instrument was that it has lesser number of items. This is certainly an advantage since few studies found that lengthy questionnaires are less likely to be completed [33, 34]. More complete responses for the questionnaire can be expected from the respondents using lesser items. The revised version of DQoL instrument has only 13 items, and hence, lesser time is needed to answer all the questions. The idea to produce a revised version is not only for patients' convenience but most importantly to strengthen the instrument.

Screening was conducted to reevaluate the content so that it can be more representative of the domains that DQoL instrument aimed to measure. This is a common process in developing a questionnaire where item development and selection are one of the critical phases to produce a valid instrument with support from stronger statistical evidence [35–37]. Reassessment has been made to all the DQoL items. There were 18 items selected for further evaluation using confirmatory factor analysis. Results from the analysis suggested that there were 13 items for the revised DQoL instrument.

Besides a shorter version, most importantly, the revised version of DQoL will be more suitable to reflect quality of life among patients with diabetes. Although validation of the revised instrument was conducted for the Malay version, the findings were supported with excellent statistical evidences. Therefore, we hope similar findings can be derived when the revised version of DQoL instrument is validated in other languages.

This study has some limitations. First of all, test-retest reliability of this questionnaire was not conducted. Future studies may consider to test the concurrent validity of the revised version of DQoL instrument using other questionnaires. Besides that, researchers are unsure to what extent the shorter version of DQoL instrument is sensitive in predicting future outcomes such as hospitalization due to diabetes complications and this too warrants future studies to be conducted.

## 5. Conclusion

In conclusion, a revised version of DQoL instrument that maintains the conceptualization of satisfaction, impact, and worry domains with only 13 items was successfully developed. Future studies should be conducted to validate the revised version of DQoL instrument in other languages.

## Figures and Tables

**Table 1:** The evaluation of the original DQoL items based on content validity, missing values and issue of redundancy.

Domain/items	Missing/“does not apply” > 10%	Duplicate	Decision
Domain: satisfaction			
*(1 = very satisfied; 2 = moderately satisfied; 3 = neither; 4 = moderately dissatisfied; 5 = very dissatisfied)* “How satisfied are you”			
(S5) … with the flexibility you have in your diet?	No	Yes (I9)	Drop
(S6) … with the burden your diabetes is placing on your family?	No	Yes (I5)	Drop
(S8) … with your sleep?	No	Yes (I6)	Drop
(S9) … with your social relationships and friendships?	No	Yes (I7)	Drop
(S10) … with your sex life?	Yes	No	Drop
(S11) … with your work, school, and household activities?	Yes	Yes (I13, W6)	Drop
(S13) … with the time you spend exercising?	No	Yes (I12)	Drop
(S14) … with your leisure time?	No	Yes (I15)	Drop

Domain: impact			
*(1 =never; 2 = very seldom; 3 = sometimes; 4 = often; 5 = all the time)* “How often do”			
(I3) … you have low blood sugar?	No	No	Drop
(I10) … your diabetes interfere with your sex life?	Yes	Yes (S10)	Drop
(I11) … your diabetes keep you from driving a car or using a machine (e.g., a typewriter)?	Yes	No	Drop
(I14) … you find yourself explaining what it means to have diabetes?	No	Yes (I16)	Drop
(I16) … you tell others about your diabetes?	No	Yes (I14)	Drop
(I19) … you find that you eat something you should not rather than tell someone that you have diabetes?	No	No	Drop
(I20) … you hide from others the fact that you are having an insulin reaction?	Yes	No	Drop

Domain: worry			
*(0 = does not apply; 1 = never; 2 = very seldom; 3 = sometimes; 4 = often; 5 = all the time)* “How often do you worry about whether you”			
(W1) … will get married?	Yes	No	Drop
(W2) … will have children?	Yes	No	Drop
(W3) … will not get a job you want?	Yes	No	Drop
(W4) … will be denied insurance?	Yes	No	Drop
(W5) … will be able to complete your education?	Yes	No	Drop
(W6) … will miss work?	Yes	Yes (S11, I13)	Drop
(W7) … will be able to take a vacation or a trip?	Yes	No	Drop
(W11) … will have someone who will not go out with you because you have diabetes?	Yes	No	Drop

**Table 2:** Evaluation of the revised version of DQoL: results from exploratory factor analysis and internal consistency.

Items		Satisfaction	Impact	Worry
S1	Time takes to manage diabetes	0.774		
S2	Time spend getting checkups	0.903		
S3	Time it takes to determine the sugar level	0.820		
S4	Current treatment	0.866		
S7	Knowledge about diabetes	0.758		
S12	Body appearance	0.482		
S15	Life in general	0.792		
I1	Feel pain associated with the treatment		0.849	
I2	Embarrassed with deal with diabetes in public		0.372	
I4	Feel physically ill		0.787	
I5	Interfere with the family life		0.609	
I6	Bad night's sleep		0.431	
I7	Limiting social relationships and friendships		0.358	
I9	Feel restricted by diet		0.413	
I18	Go bathroom more than others		0.414	
W8	Pass out			0.630
W9	Body looks differently			0.731
W10	Get complications			0.447

Extraction method: principal axis factoring. Rotation method: promax with Kaiser normalization.

**Table 3:** Summary of statistics for item and person parameters and unidimensionality using the principal component analysis (PCA) of the DQoL by Rasch analysis.

Domain	Number of items	Item^a^		Person^a^		Cronbach's alpha	Variance in data explained by measures		Unexplained variance in contrast 1 of PCA of residuals (eigenvalue)
		Separation index	Reliability	Separation index	Reliability		Empirical	Modelled	
Original version									
Satisfaction	15	6.01	0.97	2.91	0.89	0.95	55.4%	55.8%	7.7% (2.6)
Impact	20	11.58	0.99	1.69	0.74	0.85	39.7%	43.1%	8.1% (2.7)
Worry	11	12.44	0.99	1.72	0.75	0.85	48.3%	54.0%	17.8% (3.8)
Simplified/revised version									
Satisfaction	6	3.44	0.92	2.27	0.84	0.93	63.3%	63.5%	9.8% (1.6)
Impact	4	7.27	0.98	1.22	0.60	0.79	51.9%	52.4%	19.0% (1.6)
Worry	3	10.16	0.99	1.14	0.57	0.75	62.6%	62.4%	20.6% (1.7)

^a^Summary of nonextreme measured person/item. An acceptable value for reliability is >0.7, while for separation indices is >2.0. The variance explained by the measures for the empirical calculation was almost identical to the model and >40%, and the unexplained variance explained by the first contrast < 15% (eigenvalue < 2.0) suggests unidimensionality of the scale.

**Table 4:** Item fit statistics of the simplified/revised DQoL from Rasch analysis.

Domain	Item	Point measure correlation	Infit MNSQ (ZStd)	Outfit MNSQ (ZStd)
Satisfaction	(S1) Time takes to manage diabetes	0.84	0.97 (−0.4)	0.96 (−0.5)
	(S2) Time spend getting checkups	0.85	0.78 (−3.4)	0.70 (−4.2)
	(S3) Time it takes to determine the sugar level	0.85	0.91 (−1.3)	0.90 (−1.4)
	(S4) Current treatment	0.84	0.90 (−1.5)	0.81 (−2.6)
	(S7) Knowledge about diabetes	0.81	1.23 (3.1)	1.25 (3.3)
	(S15) Life in general	0.81	1.21 (2.9)	1.23 (2.9)

Impact	(I1) Feel pain associated with the treatment	0.76	1.05 (0.8)	1.04 (0.6)
	(I4) Feel physically ill	0.80	0.87 (−1.9)	0.88 (−1.8)
	(I5) Interfere with the family life	0.79	0.95 (−0.7)	0.91 (−1.2)
	(I7) Limiting social relationships and friendships	0.70	1.20 (2.6)	1.07 (0.8)

Worry	(W8) Pass out	0.74	1.27 (3.3)	1.22 (2.7)
	(W9) Body looks differently	0.84	0.83 (−2.4)	0.81 (−2.8)
	(W10) Get complications	0.87	0.92 (−1.1)	0.91 (−1.3)

MNSQ: mean square; ZStd: standardized fit statistic. Point measure correlation within 0.40 and 0.85 suggests that the items are interrelated within the domain. MNSQ range of 0.5 to 1.5 and ZStd of ±2.0 suggests an acceptable fit.

**Table 5:** Evaluation of the revised version of DQoL; result from confirmatory factor analysis and internal consistency.

Item		Factor loading	CR (95% CI)
S1	How satisfied are you with the amount of time it takes to manage your diabetes? *Sejauh manakah anda berpuas hati dengan jumlah masa yang digunakan untuk menguruskan diabetes anda?*	0.823	0.922 (0.909–0.936)
S2	How satisfied are you with the amount of time you spend getting checkups? *Sejauh manakah anda berpuas hati dengan jumlah masa yang anda gunakan untuk mendapatkan pemeriksaan doktor?*	0.879	
S3	How satisfied are you with the time it takes to determine your sugar level? *Sejauh manakah anda berpuas hati dengan jumlah masa yang anda ambil untuk menentukan paras gula anda?*	0.831	
S4	How satisfied are you with your current treatment *Sejauh manakah anda berpuas hati dengan rawatan anda sekarang?*	0.847	
S7	How satisfied are you with your knowledge about your diabetes? *Sejauh manakah anda berpuas hati dengan pengetahuan anda tentang penyakit diabetes?*	0.749	
S15	How satisfied are you with life in general? *Sejauh manakah anda berpuas hati dengan kehidupan anda secara keseluruhannya?*	0.757	

I1	How often do you feel pain associated with the treatment for your diabetes? *Berapa kerapkah anda mengalami rasa sakit yang ada kaitannya dengan rawatan diabetes anda?*	0.650	0.781 (0.745–0.818)
I4	How often do you feel physically ill? *Berapa kerapkah anda berasa sakit dari segi fizikal?*	0.693	
I5	How often does your diabetes interfere with your family life? *Berapa kerapkah diabetes menganggu kehidupan keluarga anda?*	0.764	
I7	How often do you find your diabetes limiting your social relationships and friendships? *Berapa kerapkah anda mendapati penyakit diabetes anda menghadkan hubungan social dan persahabatan anda?*	0.630	

W8	How often do you worry about whether you will pass out? *Berapa kerapkah anda bimbang yang anda akan pengsan?*	0.603	0.794 (0.755–0.832)
W9	How often do you worry that your body looks different because you have diabetes? *Berapa kerapkah anda bimbang yang tubuh badan anda nampak lain kerana menghidap diabetes?*	0.881	
W10	How often do your worry that you will get complications from your diabetes? *Berapa kerapkah anda bimbang sama ada anda akan mendapat komplikasi akibat diabetes anda?*	0.734	

**Table 6:** Comparison of fit indices for measurement model with 46 items and 15 items.

Fit indices	Cut-off value	46 items	13 items
CF fit	*p* value > 0.05	<0.001	0.528
RMSEA (95% CI)	<0.05	0.069 (0.066, 0.071)	0.049 (0.039, 0.060)
SRMR	≤0.08, good fit	0.143	0.037
CFI	≥0.95, good fit	0.772	0.966
TLI	≥0.95, good fit	0.761	0.958

Note: RMSEA: root mean square error of approximation; SRMR: standardized root mean square; CFI: comparative fit index; CI: interval.

**Table 7:** The proposed scoring for each domain and total score for a revised DQoL.

Domains	Number of items	Range of score for each item	Range of score	Converted to percentage
Satisfaction (S)	6	1 to 5	6–30	(S)/30 × 100
Impact (I)	4		4–20	(I)/20 × 100
Worry (W)	3		3–15	(W)/15 × 100
Total	13		13–65	Total/65 × 100

Higher score indicates poorer quality of life.

## Data Availability

The data used to support the findings of this study are available from the corresponding author upon request.

## Abbreviations

DQoL: Diabetes quality of life
DCCT: Diabetes Control and Complications Trial.

## Supplementary Materials

Supplementary Materials Supplementary material contains the Revised Version of DQOL (RV-DQOL13) questionnaire with 13 items.
