# Supplementary material for: A Revised Version of Diabetes Quality of Life Instrument Maintaining Domains for Satisfaction, Impact, and Worry
Source: J Diabetes Res. 2018 Jul 22;2018:5804687. doi: 10.1155/2018/5804687 (PMC6169225; doi:10.1155/2018/5804687)
Supplement: Supplementary Materials — Supplementary material contains the Revised Version of DQOL (RV-DQOL13) questionnaire with 13 items. [file 5804687.f1.pdf]

The Revised Version of DQOL (RV-DQOL13) Questionnaire

Please read every statement carefully and circle the number that best describe your feeling or situation.
Sila baca setiap penyataan dengan teliti dan tandakan nombor yang paling sesuai untuk anda..

Satisfaction Domain
Tahap kepuasan

		**Very satisfied** *Sangat Puas hati*	**Moderately satisfied**	**Neither satisfied nor dissatisfied**	**Moderately dissatisfied**	**Very dissatisfied** *Sangat tidak puas hati*
1	How satisfied are you with the amount of time it takes to manage your diabetes? *Sejauh manakah anda berpuas hati dengan jumlah masa yang digunakan untuk menguruskan diabetes anda?*	1	2	3	4	5
2	How satisfied are you with the amount of time you spend getting checkups? *Sejauh manakah anda berpuas hati dengan jumlah masa yang anda gunakan untuk mendapatkan pemeriksaan doctor?*	1	2	3	4	5
3	How satisfied are you with the time it takes to determine your sugar level? *Sejauh manakah anda berpuas hati dengan jumlah masa yang anda ambil untuk menentukan paras gula anda?*	1	2	3	4	5
4	How satisfied are you with your current treatment *Sejauh manakah anda berpuas hati dengan rawatan anda sekarang?*	1	2	3	4	5
5	How satisfied are you with your knowledge about your diabetes? *Sejauh manakah anda berpuas hati dengan pengetahuan anda tentang penyakit diabetes?*	1	2	3	4	5
6	How satisfied are you with life in general? *Sejauh manakah anda berpuas hati dengan kehidupan anda secara keseluruhannya?*	1	2	3	4	5

Impact Domain
Kesan penyakit diabetes

		**Never** *Tidak pernah langsung*	**Sometimes** *Sekali sekala*	**Often** *Kadang-kadang*	**Frequently** *Kerap*	**Always** *Sepanjang masa*
1	How often do you feel pain associated with the treatment for your diabetes? *Berapa kerapkah anda mengalami rasa sakit yang ada kaitannya dengan rawatan diabetes anda?*	1	2	3	4	5
2	How often do you feel physically ill? *Berapa kerapkah anda berasa sakit dari segi fizikal?*	1	2	3	4	5
3	How often does your diabetes interfere with your family life? *Berapa kerapkah diabetes menganggu kehidupan keluarga anda?*	1	2	3	4	5
4	How often do you find your diabetes limiting your social relationships and friendships? *Berapa kerapkah anda mendapati penyakit diabetes anda menghadkan hubungan social dan persahabatan anda?*	1	2	3	4	5

Worry Domain
Kebimbangan Sosial & kebimbangan Diabetes

		**Never** *Tidak pernah langsung*	**Sometimes** *Sekali sekala*	**Often** *Kadang-kadang*	**Frequently** *Kerap*	**Always** *Sepanjang masa*
1	How often do you worry about whether you will pass out? *Berapa kerapkah anda bimbang yang anda akan pengsan?*	1	2	3	4	5
2	How often do you worry that your body looks different because you have diabetes? *Berapa kerapkah anda bimbang yang tubuh badan anda nampak lain kerana menghidap diabetes?*	1	2	3	4	5
3	How often do your worry that you will get complications from your diabetes? *Berapa kerapkah anda bimbang sama ada anda akan mendapat komplikasi akibat diabetes anda?*	1	2	3	4	5

Table 1: The proposed scoring of each domain and total score for a revised DQoL

**Domains**	**No. of items**	**Range of score for each item**	**Range of score**	**Converted to percentage**
Satisfaction (S)	6	1 to 5	6 – 30	(S) / 30 x 100
Impact (I)	4		4 – 20	(I) / 20 x 100
Worry (W)	3		3 – 15	(W) / 15 x 100
Total	13		13 – 65	Total / 65 x 100

Higher score indicates poorer quality of life.
